# Modeling Years of Life Lost Due to COVID-19, Socioeconomic Status, and Nonpharmaceutical Interventions: Development of a Prediction Model

**DOI:** 10.2196/30144

**Published:** 2022-04-12

**Authors:** Jari John

**Affiliations:** 1 Institute of Political Science University of Heidelberg Heidelberg Germany

**Keywords:** COVID-19, pandemic, socioeconomic status, mortality, nonpharmaceutical interventions, prediction model, low-income status, life expectancy, public health, income groups

## Abstract

**Background:**

Research in the COVID-19 pandemic focused on the health burden, thereby largely neglecting the potential harm to life from welfare losses.

**Objective:**

This paper develops a model that compares the years of life lost (YLL) due to COVID-19 and the potential YLL due to the socioeconomic consequences of its containment.

**Methods:**

It improves on existing estimates by conceptually disentangling YLL due to COVID-19 and socioeconomic status. By reconciling the normative life table approach with socioeconomic differences in life expectancy, it accounts for the fact that people with low socioeconomic status are hit particularly hard by the pandemic. The model also draws on estimates of socioeconomic differences in life expectancy to ascertain potential YLL due to income loss, school closures, and extreme poverty.

**Results:**

Tentative results suggest that if only one-tenth of the current socioeconomic damage becomes permanent in the future, it may carry a higher YLL burden than COVID-19 in the more likely pandemic scenarios. The model further suggests that the socioeconomic harm outweighs the disease burden due to COVID-19 more quickly in poorer and more unequal societies. Most urgently, the substantial increase in extreme poverty needs immediate attention. Avoiding a relatively minor number of 4 million unemployed, 1 million extremely poor, and 2 million students with a higher learning loss may save a similar amount of life years as saving 1 million people from dying from COVID-19.

**Conclusions:**

Primarily, the results illustrate the urgent need for redistributive policy interventions and global solidarity. In addition, the potentially high YLL burden from income and learning losses raises the burden of proof for the efficacy and necessity of school and business closures in the containment of the pandemic, especially where social safety nets are underdeveloped.

## Introduction

More than 3 million people have lost their lives to COVID-19 with estimates projecting up to 5 million deaths by August 2021 [1]. To contain the spread of the virus, governments worldwide mainly relied on nonpharmaceutical interventions (NPIs). These came with a heavy socioeconomic burden, however, especially for the poor. According to International Labour Organization (ILO) estimates in 2020, 8.8% of all working hours were lost (the equivalent to 255 million full-time jobs) [2]. For 2021, the shortfall is expected to correspond to 140 million full-time jobs. Remittances to poorer countries declined substantially. Extreme poverty could increase by around 100 million people (or more than twice as many under the less strict poverty line of US $3.20 per day) [3,4]. Prolonged school closures that temporarily affected up to 1.5 billion students will depress long-term economic recovery [5]. Whether the long-term socioeconomic harm outweighs the benefit to protect health in the short-term is therefore a key question in the pandemic.

Governments justify the use of NPIs by referring to their proportionality. Three component parts define the proportionality of NPIs. The first two concern their efficacy (ie, the suitability and necessity of particular measures). They largely belong to the realm of epidemiologists and virologists, and are therefore beyond the scope of this paper [6-8]. The third meaning concerns the proportionality of NPIs in the narrower sense. It asks whether they are reasonable given the collateral damage they induce. However, in the pandemic, at least two problems complicate such an assessment. Subjective risk perceptions tend to have significant distortions, rendering public citizen assessments of proportionality of limited reliability [9-11]. More importantly, no common measure exists to compare the immediate health threat from COVID-19 to the mostly indirect long-term socioeconomic harm from NPIs. The time lag with which the socioeconomic damage is realized also means that the question of proportionality can only be answered in full sometime in the future.

Furthermore, important moral and legal concerns exist against weighing lives against lives in the pandemic [12]. This is particularly true because, rather than being a great equalizer, the pandemic has exacerbated existing inequalities, leaving the same people most exposed to health and socioeconomic risks. Any such comparison must therefore primarily aim at gauging the need for proportional socioeconomic compensation and raising the burden of proof for suitability and necessity of NPIs, especially in the context of resource scarcity and severity of consequences (eg, extreme poverty). After all, people can be lifted from poverty but not be resurrected from the dead.

Against this background, the paper introduces a model to compare the damage to life from COVID-19 and the socioeconomic consequences of NPIs. The starting point of the considerations is that both acute infectious diseases such as COVID-19 and a low socioeconomic status (SES) may shorten an individual’s life expectancy. Accordingly, it is possibly to assess the damage due to COVID-19 and NPIs in years of life lost (YLL). YLL refers to the gap between the age of death and the age to which a person could have lived. The approach complements but is distinct to other perspectives on the pandemic such as the burden of disease and value of life. The model rather contributes to the discourse on the relationship between health inequality and social justice [13,14]. This paper targets some of the key conceptual difficulties when attributing YLL to individual causes. Any such assessment can only be plausible estimates at best because validation of the model would require information on the share of the current socioeconomic fallout that will become permanent. Thus, the efforts at quantifying the model primarily serve for purposes of illustration.

## Methods

The model starts from the basic assumption that proportionality can be expressed as a correspondence of YLL due to COVID-19 and the socioeconomic damage from the NPIs.




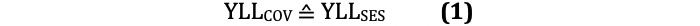




### YLL Due to COVID-19 and SES

The first part of the section discusses the first part of the equation, that is YLL due to COVID-19. The second part of the section discusses the YLL due to SES.

The analysis takes as the starting point the only large cross-country evaluation of YLL due to COVID-19 spanning 81 countries. Pifarré i Arolas et al [15] estimated the global average per COVID-19 deaths at 16 YLL. If countries are grouped along the World Bank income group classification, the average for high-income countries in their estimate was at around 13 YLL and 19 YLL for middle- and low-income countries. The estimate is based on UN World Population Prospects’ life tables for remaining life expectancy at the exact age of deaths. As the life tables are partly able to account for the systematic differences in life expectancy between global income groups, the higher YLL estimates for low- and middle-income countries reflect that COVID-19 deaths tend to occur at younger ages than in high-income countries.

However, for several reasons the life tables do not reflect the actual years a person would have lived had they not died of COVID-19. To date, no single methodology for estimating YLL exists, but it is common practice to use life tables that either assume an ideal life expectancy in a counterfactual disease- and poverty-free egalitarian world or draw on hazard ratios within the age bracket of the birth cohort [16,17]. As a result, the higher one moves in the age brackets of the life table, the more it reflects the life expectancy of the rich and healthy share of the population. The tables thus state an aspiration rather than providing information about the actual number of years an individual would have lived in the absence of a specific cause of death.

Although such a normative approach is defendable for idealistic reasons, it has weaknesses in correctly attributing YLL to individual causes. YLL has the same determinants as life expectancy in general. The question therefore is to what extent YLL can be attributed to the immediate cause of death (eg, COVID-19) or in fact reflect more fundamental causes. Unfortunately, it is difficult to specify the relative causal influence of fundamental factors such as genetic disposition [18] and SES [19,20], the mechanisms through which they work such as health behaviors [21-23] and morbidities (eg, chronic diseases) [24,25], and the immediate causes of death (eg, infectious diseases). Temporal and causal complexity and a lack of reliable data further complicate such estimates [26,27].

Because correcting YLL estimates for individual health factors such as genetic disposition, health behaviors, and comorbidities entails extraordinary data requirements, it is unsuitable for most studies. It is suggested here that a still challenging but more viable strategy is correcting for socioeconomic differences in life expectancy. In other words, dropping the assumption of an egalitarian society and accounting for socioeconomic differences in life expectancy. This may improve YLL estimates in at least three ways: a potentially more precise estimate of the actual YLL while retaining the normative claim of not accepting a lower than ideal life expectancy, a more accurate attribution of YLL to its fundamental and immediate causes, and consequently better policy advice tailored to specific health and socioeconomic vulnerabilities. To that end, country-specific findings on socioeconomic differences in life expectancy should be combined with data on the socioeconomic profile of COVID-19 deaths (or where such data is lacking seroprevalence and hospitalization rates).

Socioeconomic differences in life expectancy in high-income countries usually amount to 5 to 10 years between groups with a low and high SES (eg, between the first and fifth quintile of the income distribution) [28]. They may, however, reach up to 15 to 20 years in poorer and more unequal societies or when using more fine-grained indicators of SES [19,29,30]. In the United Kingdom, for example, a country that collects relatively detailed socioeconomic data, life expectancy differs by 7.8 years between the first and the fifth quintile (and 9.4 years between the first and the highest decile) of the Index of Multiple Deprivation [31]. In Germany, differences in life expectancy between people with a low SES and those with a high SES is around 6.6 years [32]. Comparable data for low- and middle-income countries is scarce but higher overall life span variability and the leveling effect of socioeconomic progress suggest an even larger socioeconomic gap in life expectancy [33,34]. Brazil, for example, an upper middle–income country with persistent and high socioeconomic inequality was able to reduce life span inequality from 19 to 12 years between 1991 and 2010 with socioeconomic development explaining the vast majority of this development [35]. In many low- and lower middle–income countries, the lowest quintile of the income distribution often lives below the poverty line, which may result in even higher inequality in life expectancy [30]. Amid existing data uncertainties, this model assumes a socioeconomic gap in life expectancy between people with low and high SES of 5 to 15 years.

Regarding the socioeconomic distribution of COVID-19 deaths, studies consistently find that people with a low SES are significantly overrepresented. The United Kingdom reports the most credible data on socioeconomic deprivation. Here, the most deprived quintile accounts for 23% of COVID-19–related deaths [36]. In the United States, the poorest quintile has one-third more comorbidities, twice the case count and death rate, and accounts for one-third of COVID-19–related deaths (people with below median income account for two-thirds) [37,38]. Swedish data from the early periods of the pandemic put the share of deaths with a low SES at even 40% [39]. In Germany and Scotland, people with a low SES account for 40% and 50% of hospitalizations, respectively [40,41]. Awaiting relevant data from low- and middle-income countries, the model assumes a range of 20% to 40% for COVID-19 deaths with a low SES (Table 1).

**Table 1:** ∅ years of life lost due to socioeconomic status (SES).

Distribution of deaths by SES group (low/mid/high; %)	Socioeconomic gap in life expectancy (years)				
	5	7.5	10	12.5	15
20/60/20	2.5	3.8	5	6.3	7.5
30/60/10	3	4.5	6	7.5	9
40/50/10	3.3	4.9	6.5	8.1	9.8

Because the life tables for the COVID-19–YLL previously discussed assume that everyone is rich (and healthy), the average YLL per person must be corrected for the combined effect of the socioeconomic gap in life expectancy and the socioeconomic distribution of COVID-19 deaths. Table 1 summarizes the stylized findings across various scenarios.

For example, in a country like the United Kingdom where approximately 23% of COVID-19 deaths have a low SES and 60% a medium SES, and the socioeconomic gap in life expectancy is around 7.8 years, the 11.2 YLL estimated in Pifarré i Arolas et al [15] would need to be corrected downward by 4.1 YLL to 8.1 YLL. In other words, 4.1 YLL do not occur due to COVID-19 but can be attributed to a low SES:




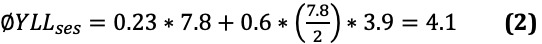




For a country with a distribution of 40% and 50% with low and middle SES, respectively, and a socioeconomic gap in life expectancy of 15 years, the YLL estimate due to COVID-19 would have to be corrected downward by 9.8 YLL. Such distribution may be more likely in low- and lower middle–income countries where the informal economy accounts for 50% to 90% of employment. Together with informal housing (ie, slums), this is an important driver of COVID-19 incidence and deaths [42-44]. The correction would reduce the average 19 YLL due to COVID-19 in this income group to 9.2 YLL.

In sum, the idealistic estimates from the UN life tables of 12 to 19 YLL need to be corrected downward by around 25% to 50%, depending on how egalitarian the life expectancy and distribution of COVID-19 deaths are in a country. This correction only partially accounts for comorbidities, which are indeed lower but not absent in people with a high SES. Correcting for comorbidities in previous studies resulted in a further reduction by 1 to 3 YLL [45]. To account for this, the model uses 8 YLL per COVID-19 deaths as its standard parameter and 6 YLL and 10 YLL as an alternative specification.

Six hypothetical scenarios (W1-6) with different numbers of COVID-19 deaths are constructed (see Table 2). Current empirical projections for the global pandemic estimate up to 6 million deaths by December 2021 and twice as many excess deaths [1]. With slow vaccine rollout in most parts of the world and uncertain protection against new virus variants, it cannot be ruled out that this number multiplies over the following years. Additionally, a less stringent global response or a more deadly virus could have yielded substantially higher numbers of death.

**Table 2:** Total years of life lost (YLL) due to COVID-19.

Scenario	COVID-19 deaths (millions)	Average YLL per COVID-19 death (millions)		
		∅6	∅8	∅10
W1	5	30	40	50
W2	7.5	45	60	75
W3	10	60	80	100
W4	30	180	240	300
W5	50	300	400	500
W6	70	420	560	700

### YLL Due to NPIs

The model also draws on the socioeconomic gap in life expectancy to ascertain the potential YLL from loss in SES (eg, due to unemployment or forgone education). However, it would almost certainly be an overestimate to infer that a loss in SES group directly translates into an equivalent reduction of the individual life span. As previously noted, a host of factors determine life expectancy, which means that only a part of it is in fact malleable. While exact causal weights are still to be determined, the model can draw on a number of studies that made considerable headway into estimating the individual contribution of factors such as income and education to the socioeconomic gap in life expectancy. In the European mean, low income explains around 10% to 20% of an average 5-year gap in life expectancy between educational groups [46]. For disability-adjusted life expectancy, it is around 20% of an 8.5-year gap in life expectancy between low and high educational groups [47]. Educational and occupational status also account for around 20% of the 10-year gap in life expectancy between SES groups [48,49]. Taken together, the model therefore assumes that key SES factors such as income and educational status may each account for about 20% of the socioeconomic gap in life expectancy.

To date, little credible data exists that could confirm whether these findings can travel easily from European high-income countries to the rest of the world. There are reasons to believe that socioeconomic determination of life expectancy is higher in low- and middle-income countries. In poorer countries, morbidity and mortality are generally higher, but health behaviors account for a smaller share of the socioeconomic differences in life expectancy [25,50]. Education also tends to entail higher-income premiums [51] but, like health services, is often not universally supplied and depends on personal income. While this is unlikely to reflect exact causalities, the Socio-Demographic Index of the Global Disease Burden Project accounts for 85% of international differences in average healthy life expectancy by building the geometric mean of lagged per capita income, education of the population aged ≥15 years, and the fertility rate of women aged ≥25 years (as a proxy for the standing of women in society) [52]. Against this background, it seems plausible that in middle- and low-income countries factors such as income and education may each account for 30% and more of the socioeconomic differences in life expectancy.

Based on these findings, it is possible to construct a rough estimate of YLL due to loss in SES, depending on the size of the socioeconomic gap in life expectancy (5-15 years) and the degree of socioeconomic determination (20%-40%; Table 3). The YLL vary between 0.5 in a rather egalitarian high-income country and a 3 YLL in a highly unequal low-income country. Given that in the latter case a loss in status group often entails falling into poverty, this seems a rather conservative estimate.

**Table 3:** YLL^I,E^ (per capita) for decline in socioeconomic status.

Socioeconomic determination	Socioeconomic gap in life expectancy (years)				
	5	7.5	10	12.5	15
High income (20%)	0.5	0.8	1	1.3	1.5
Middle income (30%)	0.8	1.1	1.5	1.9	2.3
Low income (40%)	1	1.5	2	2.5	3

In the proportionality model that has been developed, the two main causes of loss in SES group and components of the socioeconomic damage (*YLL_SES_*) in the pandemic are income loss (*YLL_I_*) and forgone education (*YLL_E_*). The main factors behind *permanent* income loss are unemployment, reduction of working hours, and economic inactivity. Forgone education may result from unrealized secondary or tertiary education due to dropping out or a lack of qualification or financial means for higher education:




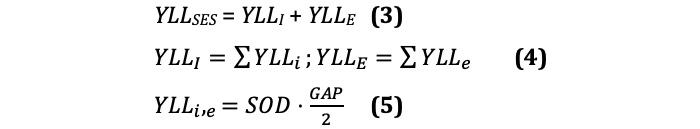




Educational loss in the pandemic further differentiates in two components: the most unfortunate cases where income loss or temporary school closures result in students permanently forgoing a higher educational bloc depriving them of secondary or tertiary education (YLLe1) and the average income losses from school closures that affect the vast majority of students (YLLe2). Past examples show that even short episodes of temporary school closures have a measurable average impact on income in later life. The first pandemic-related school closures may reduce lifetime earnings by 1% to 4%, depending on the subsequent ability for learning compensation [53,54]. Adding the second round of school closures at the turn of the year 2020/2021 and considering that longer closures add exponentially, current losses in lifetime earnings may amount to 2% to 8%. For simplicity, the model assumes an average of 5% reduced lifetime earnings (or about one-eighth of a decline in SES group).




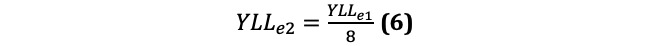




For many low- to middle-income countries, this is likely to be an underestimate, given that school closures were on average longer and entail higher dropout rates and higher income premiums [55,56].

### Proportionality Model

In the proportionality model the loss of life years in these scenarios is then juxtaposed with the socioeconomic damage in the pandemic. The main idea is to calculate the number of people for which the loss in SES would have to become permanent for the amount of YLL to be equivalent.




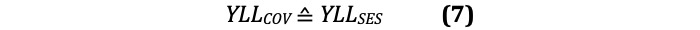




To that end, the individual components of socioeconomic damage are distributed among subgroups of the globally affected population (Ng):

*N_g_*= *n_e_* + *n_i_* + *n_p_*
**(8)**

- Workers(n_i_i) ≈ 3,492,000,000 (from ILO)
- Students(n_e_e) ≈ 1,500,000,000 (from UNESCO)
- Extremelypoor(n_p_) ≈ 640,000,000 (World Bank)

Learning loss is divided into two subgroups. Those students with average learning and subsequent income loss from school closures YLLe2 and the worst hit students that forgo a 3- to 4-year higher learning block YLLe1 (ie, additional dropouts due lack of funding or qualification for higher education). Because students with a high SES may have more capacities to compensate for learning losses, it is assumed that two-thirds of the students worldwide (0.9 billion) had average learning and subsequent income loss due to school closures.

*YLL_E_*_2_ = 0.66 ⋅ *n_e_* ⋅ *YLL_e_*_2_  **(9)**

The resulting value is subtracted from the overall YLL due to COVID-19. The remaining damage is then distributed among the students with a learning block loss (YLL_E1_), people with an income loss (YLL_I_), and those that fall into extreme poverty as a result (YLL_P_).

*YLL_COV_* – *YLL_E_*_2_ = *YLL_E_*_1_ + *YLL_I_* + *YLL_P_*  **(10)**

Each group carries a weighted burden that reflects group size and the social gradient (α,ꞵ,γ). Income losses account for slightly more than half (0.54) and forgone education (0.23) and poverty (0.22) each for slightly less than one-quarter of all YLL. For the global average, the factor of socioeconomic determination is set at 0.3 and 0.4 for the extremely poor. The average socioeconomic gap in life expectancy is set at 7.5 years.




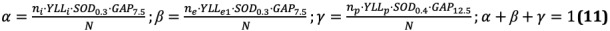




With these shares it is possible to individually calculate the total number of workers (Xi), students (Xe), and extremely poor (Xp) for whom the socioeconomic damage in the pandemic would have to become *permanent*.




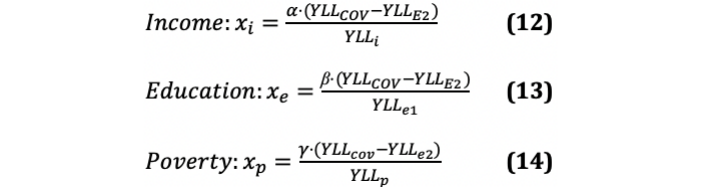




## Results

The standard model specifications aim to reflect the global average (8 YLL per COVID-19 death, a 7.5-year socioeconomic gap in life expectancy, and a socioeconomic determination factor of 0.3). Tables 4 and 5 read as follows. Each row displays the total number of workers, poor, and students for which the socioeconomic damage would have to become *permanent* for the YLL to be equivalent of those attributable to COVID-19. Values are negative when the average socioeconomic damage from school closures (YLLe2) is higher than the YLL due to COVID-19. A separate column on the right provides the common percentage share, which by definition is identical for all groups (eg, 1% of all workers, extremely poor, and students). Because the YLL from YLLe2 alone amount to an equivalent of approximately 20 million COVID-19 deaths, although the individual burden distributed over 900 million students is relatively small, a second table displays the outcomes excluding YLLe2.

**Table 4:** Equivalent permanent socioeconomic damage with standard specifications.

Scenario	COVID-19 deaths (millions)	∅8 years of life lost			
		Income loss (millions)	Extremely poor (millions)	Education loss (millions)	Share (%)
W1	5	–47.9	–8.8	–20.6	–1.4
W2	7.5	–38.3	–7.1	–16.4	–1.1
W3	10	–28.6	–5.3	–12.3	–0.8
W4	30	48.7	9	20.9	1.4
W5	50	126	23.2	54.1	3.6
W6	70	203.3	37.5	87.3	5.8

**Table 5:** Equivalent socioeconomic damage (excluding school closures).

Scenario	COVID-19 deaths (millions)	∅8 years of life lost			
		Income loss (millions)	COVID-19 poor (millions)	Education loss (millions)	Share (%)
W1	5	19.3	3.6	8.3	0.6
W2	7.5	29	5.3	12.5	0.8
W3	10	38.7	7.1	16.6	1.1
W4	30	116	21.4	49.8	3.3
W5	50	193.3	35.6	83	5.5
W6	70	270.6	49.9	116.2	7.7

To put the model estimates in perspective, current projections by major international organizations can contextualize the findings. It should be noted, however, that the main outcomes of interest will only be available years if not decades from now because, to have a significant effect on life expectancy, a loss in SES has to become permanent. Regarding education loss, the negative values in the first three rows of Table 5 suggest that YLL from temporary school closures outweighs the YLL due to COVID-19 in the currently most probable scenarios. Only in scenarios W4-6 would an increase in students that will forgo a whole 3- to 4-year educational block be proportional (eg, because of them not qualifying for further education or dropping out into the labor market is uncertain). Table 6 shows that, even excluding the effect of YLLe2, the socioeconomic damage in scenarios W1-3 is likely to be disproportionate to the YLL due to COVID-19. Already in September 2020, UNESCO warned that at least 24 million students could drop out of school due to school closures (>W3) [57]. One year later, 168 million students worldwide have missed out on learning for almost an entire year, and another 214 million missed more than 9 months. For 140 million children, the first day of school has been indefinitely postponed [58]. One in three countries is not taking measures to compensate for learning losses [59]. The number of children that will forgo a whole education bloc is thus likely to be above even the worst-case scenarios. Another way to put it is that children are bearing a disproportionate share of the socioeconomic consequences of NPIs.

**Table 6:** Proportional socioeconomic damage across different model specification.

	SOD^a^: 0.2 and ∅YLL^b^: ∅6				SOD: 0.3 and ∅YLL: ∅8				SOD: 0.5 and ∅YLL: ∅10		
	GAP^c^: 5	GAP: 7.5	GAP: 10	GAP: 7.5		GAP: 10	GAP: 12.5	GAP: 10		GAP: 12.5	GAP: 15
W1	–3.4%	–2.3%	–1.7%	–1.4%		–1.0%	–0.8%	–0.7%		–0.6%	–0.5%
W2	–2.9%	–2.0%	–1.5%	–1.1%		–0.8%	–0.7%	–0.5%		–0.4%	–0.3%
W3	–2.5%	–1.6%	–1.2%	–0.8%		–0.6%	–0.5%	–0.3%		–0.2%	–0.2%
W4	1.3%	0.8%	0.6%	1.4%		1.0%	0.8%	1.3%		1.0%	0.8%
W5	5.0%	3.3%	2.5%	3.6%		2.7%	2.2%	2.8%		2.2%	1.9%
W6	8.7%	5.8%	4.4%	5.8%		4.4%	3.5%	4.4%		3.5%	2.9%

^a^SOD: socioeconomic determination of life expectancy.

^b^YLL: year of life lost.

^c^GAP: socioecomonic difference in life expectancy.

Another group bearing an even more disproportionate share of the pandemic burden are the extremely poor. The World Bank estimates their number has increased by approximately 100 million [3]. As the impact of the pandemic continues to worsen in low- and lower middle–income countries, the initial increase is expected to persist. This is three and two times the proportional YLL damage in the worst-case scenarios of Tables 4 and 5, respectively (>W6). Income loss is also likely to remain disproportionate even if the long-term loss in jobs will settle significantly below 100 million (>W3/W4). The ILO estimates that in 2020 working hours equivalent of 255 million full-time jobs were lost [60]. In 2021, working hours will still be 4.4% below the projection for the no-pandemic scenario. Lower middle–income countries experienced the strongest decline. The 2020 estimate divides into 30 million people affected by forgone job growth, 131 million by reduced working hours, and 114 million by employment loss. The last group is divided into 33 million unemployed and 81 million people economically inactive of which the latter are unlikely to recover anytime soon, if ever.

For a second set of results, the standard model specifications were adapted to reflect different country conditions (see Table 6). The three columns on the left assume conditions more similar to a typical high-income country. The factor of socioeconomic determination is set at 20%; the average per capita YLL due to COVID-19 at 6 YLL; and the socioeconomic gap in life expectancy varies between 5 YLL (South Europe), 7.5 YLL (Central, Western Europe), and 10 YLL (Eastern Europe, United States). The three middle columns show two variations of the standard specification with a higher socioeconomic gap in life expectancy (10 and 12.5 years) to account for more unequal and lower-income countries. The three columns on the right assume conditions that may be more characteristic of low-income countries with a higher level of socioeconomic determination (40%), a high loss of per capita life years due to COVID-19 (10 YLL), and a wide socioeconomic gap in life expectancy (10-15 years). Again, two tables were produced including and excluding YLLe2. For reasons of readability, the tables present the changing values only as the common percentage share of people affected with income loss, extreme poverty, and foregone education.

The different model specifications obtain two main results. One at the cross-country level and one at the within-country level. First, the differences in parameters tend to largely even out across the different model specifications. The first three scenarios remain disproportionate in low-, middle-, and high-income countries, meaning the socioeconomic fallout outweighs the COVID-19–related YLL. The results also largely hold when dropping the average damage from school closures (YLLE2; Table 7). Larger differences only occur in scenario W4-6. At the extreme ends of the model specifications, the proportionality of the socioeconomic damage differs by a factor of 3 (2.9%-8.7%), reflecting the steeper social gradient in low-income countries. The second main result is that, at constant YLL per COVID-19 death, the socioeconomic damage becomes disproportionate much faster in more unequal societies. In more egalitarian high-income countries, twice the socioeconomic damage is proportional than in the most unequal ones. In middle- and low-income countries, these differences are less pronounced but remain significant.

**Table 7:** Proportional socioeconomic damage across different model specification (excluding school closures).

	SOD^a^: 0.2 and ∅YLL^b^: ∅6				SOD: 0.3 and ∅YLL: ∅8				SOD: 0.4 and ∅YLL: ∅10		
	GAP^c^: 5	GAP: 7.5	GAP: 10	GAP: 7.5		GAP: 10	GAP: 12.5	GAP: 10		GAP: 12.5	GAP: 15
W1	0.9%	0.6%	0.5%	0.6%		0.4%	0.3%	0.4%		0.3%	0.3%
W2	1.4%	0.9%	0.7%	0.8%		0.6%	0.5%	0.6%		0.5%	0.4%
W3	1.9%	1.2%	0.9%	1.1%		0.8%	0.7%	0.8%		0.6%	0.5%
W4	5.6%	3.7%	2.8%	3.3%		2.5%	2.0%	2.3%		1.9%	1.6%
W5	9.3%	6.2%	4.7%	5.5%		4.2%	3.3%	3.9%		3.1%	2.6%
W6	13.1%	8.7%	6.5%	7.7%		5.8%	4.6%	5.4%		4.4%	3.6%

^a^SOD: socioeconomic determination of life expectancy.

^b^YLL: year of life lost.

^c^GAP: socioecomonic difference in life expectancy.

## Discussion

### Principal Results

This paper sets out to narrow in on the difficult question of proportionality between the health and socioeconomic fallout in the pandemic. To do so, it first made the case that dropping the assumption of a poverty-free and egalitarian society can make estimates of YLL due to COVID-19 about 25% to 50% more accurate. To put it differently, up to half of the YLL extracted from life tables may in fact be socioeconomic differences in life expectancy. Because SES is associated with morbidity and mortality more generally, the approach may yield analytic benefits beyond the current pandemic. Ecological data of the SES for the population of interest may partly proxy for a lack of individual-level data on the prevalence of morbidity and other risk factors.

The application to the pandemic highlights the difficult trade-offs involved in the short- and long-term protection of health. While NPIs target immediate health concerns, the long-term socioeconomic damage is likely to entail a steep cost to life, especially among the poor and children, that requires immediate attention in the aftermath of the pandemic. In countries that lack the necessary resources to compensate for the socioeconomic damage in the pandemic, more drastic NPIs such as business and school closures should only be implemented as a last resort. The tentative results further suggest that avoiding a relatively minor number of 4 million people with income loss, 1 million extremely poor, and 2 million students with a higher learning loss can save a similar amount of life years as saving 1 million people from dying from COVID-19. The extent of the socioeconomic damage further suggests that decision makers took measures in expectancy of a worst-case scenario (W6). Interestingly, the question of proportionality has otherwise been rather similar across different income groups, largely because the social gradient and the associated loss of life is steeper for both COVID-19 and the NPIs. Levels of within-country inequalities may, however, be a key concern for estimating the proportionality of the NPIs. This is especially true because a wider socioeconomic gap in life expectancy signals a weaker social safety net that could compensate for losses.

### Limitations

The approach comes with a number of important limitations. The assumptions on the extent of socioeconomic determination of the life expectancy and the size of the socioeconomic gap in life expectancy in low- and middle-income countries require a more thorough basis in empirical data that to date is missing. Furthermore, the model does not account for the nonlethal health impacts in the pandemic (eg, “Long-Covid,” psychosocial harm, or overwhelmed hospitals). Future research could include such information using quality-adjusted estimates such as the healthy life expectancy. The model also does not account for the COVID-19–related disease burden on economic activity. Issues of the relative causal weight of NPIs have been largely put aside. Harsher NPIs are sometimes invoked to justify reducing the socioeconomic damage in the pandemic. However, existing research into the relationship has thus far been unable to disentangle the causal role of voluntary behavioral change, formal and informal NPIs, and the objective disease burden in reducing economic activity. NPIs may only account for about one-third of the variation in COVID-19 mortality and around 20% of reduced business activity [8,61]. Against this background, the model has assumed that causal uncertainties on both sides of the equation may eventually even out. Future research should carefully assess issues of causal weight and direction.

### Conclusions

The application to the pandemic highlights the difficult trade-offs involved in the short- and long-term protection of health. While NPIs target immediate health concerns, the long-term socioeconomic damage is likely to entail a steep cost to life, especially among the poor and children, that requires immediate attention in the aftermath of the pandemic. In countries that lack the necessary resources to compensate for the socioeconomic damage in the pandemic, more drastic NPIs such as business and school closures should be weighed carefully.

## Abbreviations

ILO: International Labour Organization
NPI: nonpharmaceutical intervention
SES: socioeconomic status
YLL: years of life lost
